# Reduction of Movement in Neurological Diseases: Effects on Neural Stem Cells Characteristics

**DOI:** 10.3389/fnins.2018.00336

**Published:** 2018-05-23

**Authors:** Raffaella Adami, Jessica Pagano, Michela Colombo, Natalia Platonova, Deborah Recchia, Raffaella Chiaramonte, Roberto Bottinelli, Monica Canepari, Daniele Bottai

**Affiliations:** ^1^Department of Health Science, University of Milan, Milan, Italy; ^2^Department of Molecular Medicine, University of Pavia, Pavia, Italy

**Keywords:** neural stem cells, motor inactivity, neurodegenerative diseases, astronauts, hindlimb unloading, neurogenesis, Cdk5RAP1, metabolism

## Abstract

Both astronauts and patients affected by chronic movement-limiting pathologies face impairment in muscle and/or brain performance. Increased patient survival expectations and the expected longer stays in space by astronauts may result in prolonged motor deprivation and consequent pathological effects. Severe movement limitation can influence not only the motor and metabolic systems but also the nervous system, altering neurogenesis and the interaction between motoneurons and muscle cells. Little information is yet available about the effect of prolonged muscle disuse on neural stem cells characteristics. Our *in vitro* study aims to fill this gap by focusing on the biological and molecular properties of neural stem cells (NSCs). Our analysis shows that NSCs derived from the SVZ of HU mice had shown a reduced proliferation capability and an altered cell cycle. Furthermore, NSCs obtained from HU animals present an incomplete differentiation/maturation. The overall results support the existence of a link between reduction of exercise and muscle disuse and metabolism in the brain and thus represent valuable new information that could clarify how circumstances such as the absence of load and the lack of movement that occurs in people with some neurological diseases, may affect the properties of NSCs and contribute to the negative manifestations of these conditions.

## Introduction

Several neurological diseases are associated with, or are the cause of, movement impairments; among them, spinal cord injury, multiple sclerosis, and spinal muscular atrophy are examples with analogous effects on anti-gravity muscles. Similarly, it is well known that prolonged space missions and extended bed-rest induce functional alterations in many organs of the human body, including modifications of skeletal neuromuscular function (Kakurin et al., 1972) due to anti-gravity muscle reduced activity.

While the relationship between physical activity and cognitive ability has been known for centuries, recent studies demonstrate the significant impact that voluntary physical activity exerts on neurogenesis (van Praag et al., 1999; Adami and Bottai, 2016). Voluntary physical activity can both produce a significant increase in the levels of proliferative progenitor cells and restore neurogenesis artificially altered in rodent models of muscle disuse/inactivity (Farioli-Vecchioli et al., 2014). Voluntary physical activity has also been shown to overcome the age-dependent depletion of hippocampal neurogenesis (van Praag et al., 2005). In contrast, there is relatively little information available about the effect of prolonged muscle disuse on neurogenesis *per se*; previous studies describe *in vivo* changes with little focus on the differentiation process (Yasuhara et al., 2007). Thus we currently lack a detailed *in vitro* study of the influence of muscle reduced activity on neural stem cells (NSCs) characteristics.

Adult neurogenesis is restricted to few areas of the mammalian brain: the sub-ventricular zone of the lateral ventricles (SVZ), where it can be detected by evaluating the proliferation capability (for instance using marker associated to the cell cycle progression such as Ki67) (Shen et al., 2008; Liu and Crews, 2017), the sub-granular zone of the dentate gyrus of the hippocampus and the spinal cord (Bottai et al., 2003).

The synergistic action of extrinsic and intrinsic factors in the microenvironment of neurogenic areas controls the fate of the NSCs and is able to adjust the balance between undifferentiated progenitor cells and newly differentiated cells (Bottai et al., 2003).

The knowledge of the determinants affecting neurogenesis in individuals with movement restrictions is of pivotal interest in the attempt to develop new strategies to reduce the negative central and peripheral impact of motor deprivation in immobile patients and in astronauts. The effects of prolonged motor restraint on neurogenesis and the role of trophic determinants involved in this phenomenon can be studied using a recognized rodent model of severe motor deprivation: the so-called hindlimb unloading (HU) mouse model (Morey et al., 1979; Desaphy et al., 2005) which reproduces the absence of weight support on hindlimbs. In the literature, only a few studies have shown alterations in the levels of nerve growth factor (NGF) mRNA and of the brain-derived neurotrophic factor (BDNF) in the somatosensory cortex, supporting the hypothesis that disuse regulates neurotrophic factor expression (Dupont et al., 2005). A change has also been demonstrated in learning ability and memory in rats subjected to anti-gravity (Sun et al., 2009). The central effects of HU condition include a significant decrease in hindlimb representation on the motor cortex of the rat (Langlet et al., 2012). By contrast, physical exercise such as running leads to cell cycle shortening in some progenitors, and the S-phase shortening represents a major intrinsic regulator of the proneurogenic effect in the hippocampus exerted by running (Farioli-Vecchioli et al., 2014).

Low levels of exercise are thought to represent a major risk factor of developing metabolic alteration (Laaksonen et al., 2002) that could affect the central nervous system and in particular some neurogenic areas (Bottai and Adami, 2013; Adami and Bottai, 2016). L-lactate is a common metabolite in mammals, its production occurs in all cells including neurons and glia, and lactate is used actively by brain cells in culture (Medina and Tabernero, 2005). Pyruvate is formed during glycolysis and part of it is converted into L-lactate by lactate dehydrogenase (LDH). This prompted us to study lactate production as a marker of the metabolic activity of NSCs.

Our studies provide a new line of experimental investigation that can complement previous works on the role of exercise in neurogenesis. Overall, our analysis indicates the importance of the role of movement on NSCs properties *in vitro*.

## Materials and methods

### Animal model

The experiments were performed on 4-month-old male mice of the C57BL/6 strain. To induce a motor deprivation model we used the HU model (Morey et al., 1979; Desaphy et al., 2005); which is a representation of muscle non-use characterized by muscle atrophy, and suitable for mimicking the changes noticed during spaceflight or prolonged bed rest. Briefly, the animals (housed at a temperature of between 20° and 24°C, humidity between 35 and 50% and with a light cycle of 12 h) were suspended individually in cages (built by the experimenters following the model of Wronski and Morey-Holton; Morey-Holton and Globus, 2002) by thin string tied at one end to the tail and connected at the other end to the top of the cage; the length of the string was adjusted to allow the animals to move freely on their forelimbs, while the body was inclined at 30–40° from the horizontal plane such that only the forelimbs touched the bottom of the cage. During the suspension period (14 days), the mice were supplied with food and water *ad libitum* and their weight was checked daily; the mice's state of well-being was ascertained throughout the period of suspension (the veterinarian visited the mice three times during the unloading experiment, the first, the 7th and the 14th day) (see also Supplementary Materials and Methods). On the 14th day of suspension, both groups of mice were sacrificed.

This study was carried out in strict accordance with the recommendations of the Ethics Committee for Animal Experimentation at the University of Pavia. The animal experiments were performed in conformity with the European Law Implementation of Directive 2010/63/EU of the European Parliament and with that of the Council on 22 September 2010 on the protection of animals used for scientific purposes; the Italian Ministry of Health authorized the research project (Authorization number 727/2016-PR). All efforts were made to minimize suffering in the animals; the sacrifice was performed under urethane (Carbamate) anesthesia by means of intraperitoneal injection (1.2 g/kg weight), and the mice were killed by cervical dislocation.

### Histology and immunofluorescence study of the SVZ

The brains were dissected and divided coronally to the bregma and treated as described in Bottai et al. (2014). Briefly, they were immersed in 4% paraformaldehyde at 4°C for 4 h, and rinsed three times for 5 min (min) with phosphate buffer PB; they were then placed first in 15% sucrose-phosphate buffer saline PBS for 3 h and then in 30% sucrose-PBS overnight. Specimens were embedded in optimal cutting temperature compound (OCT), frozen on dry ice and cut into 10 μm-thick transverse sections by means of a cryostat (Leica CM1510).

We performed immunofluorescence studies staining for the epitope Ki67 (ab 92353, abcam, 1:100) the sections were rinsed with PBS 1X for 15 min, incubated in NH4Cl 0.05 M for 30 min, and washed three times for 5 min with PBS 1X. For antigen retrieval samples were treated with sodium citrate 10 mM pH 6 at 95°C for 5 min, cooled down for 1 h at room temperature (RT), and washed three times for 5 min in PBS 1X. The tissue was then blocked in a solution containing 2% of normal goat serum (NGS), 1.25% bovine serum albumin (BSA) and 0.1% Triton X100 for 90 min at RT. The slices were then incubated for 48 h at 4°C in a solution of 0.75% bovine serum albumin (BSA) and 0.05% Triton X100 (incubation solution) containing the Ki67 antibody. After treatment with primary antibodies, sections were washed with PBS 1X and Triton X100 0.05% three times for 5 min and incubated for 15 min in the incubation solution. The slices were then incubated in anti-rabbit secondary antibody Alexafluor 488-conjugated (IS20012, immunological Science, USA, 1:800) solution at RT for 5 h, counterstained with 4′,6-Diamidine-2′-phenylindole dihydrochloride (DAPI) 300 nM and mounted using the FluorSave Reagent (Calbiochem). Negative controls (no primary antibody) were used to set up the background level for confocal analysis.

The counting was conducted at the boundary between the lateral ventricle and the parenchyma of the ventral part of the SVZ within 300 μm from the ventricle where the most proliferating cells are present over an area of 0.5 mm^2^. At the bregma level, two 10 μm sections per animal were analyzed, 50 μm apart, and averaged. The number of positive cells in the selected area was obtained as the average between the animal group (CTR and HU) used in the experiment. The images were acquired using the Leica TCS SP2 confocal microscope and the stacks were displayed as maximum intensity projections. On each image, we drew an area 300 μm wide and the entire length of the SVZ lateral wall by using Leica Confocal Software version 2.6, with which we also calculated the surface area.

The number of positive cells in the selected area was obtained as the average between the animals (CTR and HU) used in the experiment. The result obtained was divided by the average areas measured by Leica Confocal Software version 2.6. The area studied has an enriched presence of enriched of Ki67 since most of the proliferating cells of the sub-ventricular zone invade the parenchyma.

### Dissection of the brain and preparation of NSCs

The procedure was performed as described in Daniela et al. (2007); Bottai et al. (2008) and Givogri et al. (2008). Briefly, the brains were removed and tissues containing the SVZ were dissected out. Each culture was derived from a single mouse. The dissected tissue was maintained in a PB 0,01 M solution containing penicillin and streptomycin 100 U/ml each (Invitrogen, San Diego, CA) and glucose (0.6%) at 4°C during the dissection of the other samples; then an enzymatic dissociation at 37°C was performed (Bottai et al., 2008).

Tissues underwent centrifugation and mechanical disaggregation until single cells were obtained (Bottai et al., 2008). Finally, the supernatant was discarded and the pellet re-suspended in 5 ml of proliferation medium (PM) (Gritti et al., 2002). In these conditions, in 3–5 days NSCs present in the tissue gave rise to spheroidal structures (neurospheres) which were harvested, mechanically dissociated and replated in PM at a concentration of 10,000 cells/cm^2^.

### Proliferation assay

Growth curves were obtained from six cultures (3 CTR and 3 HU) starting from the third passage (P3). At each passage, cells were mechanically dissociated when the neurospheres reached the appropriate dimension (about 0.1 mm). They were then mechanically dissociated and plated at the density of 10,000 cell/cm^2^ in a 25 cm^2^ flask. The cumulative total number of cells for each passage was calculated multiplying the proliferation rate (viable cell harvest number/inoculum cell number) by the cumulative total number of cells of the previous passage (Bottai et al., 2012). The calculation of the population doubling time was performed using the algorithm provided by http://www.doubling-time.com.

### Differentiation assays

Forty thousand cells were plated into a 48-multiwell plate containing one 10 mm coated (Cultrex, Tema Ricerca, Italy) round glass coverslip in PM medium without EGF for 2 days, then this medium was removed and substituted by PM medium without EGF and FGF that contained 1% of fetal calf serum. Differentiation was reached after 7 days at 37°C 5% CO_2_ (Gritti et al., 2002; Bottai et al., 2008). On the seventh day, cells were washed once with PBS 1X and fixed with 4% paraformaldehyde (PFA) for 10 min at RT. The primary antibodies used were: mouse anti-β-tubulin III monoclonal antibody (1:300, Immunological Sciences AB-10288); rabbit anti-GFAP polyclonal antibody (1:300, Immunological Sciences AB-10635) and mouse anti-O4 monoclonal antibody (1:300, Immunological Sciences MAB-10259), for intracellular epitopes the cells were permeabilized with 0.1% triton X100.

The secondary antibodies conjugated with fluorophores were Alexa-fluor 488 (Goat-anti mouse Immunological Sciences IS20010) and Alexa-fluor 555 (Goat anti-rabbit Immunological Sciences IS20012) at a dilution of 1:800.

### Confocal analysis

Images were acquired using a Leica TCS SP2 microscope with 405 diode, He/Ne and Ar/Kr lasers.

### RNA isolation from proliferating neurospheres

In order to use 2^*^10^6^ cells for RNA preparation, 750,000 cells were seeded in a T75 cm^2^ flask. When spheres were of the appropriate dimension, a fraction of the culture was mechanically dissociated and counted. This step made it possible to determine the approximate number of cells per ml of medium, and the volume of culture containing 2^*^10^6^ cells was taken from the culture flask. The sphere containing medium was centrifuged at 100 gs for 10 min and re-suspended in 1,400 μl of sterile PBS 1X. Cells were transferred into a 1.5 mL RNase-free tube and centrifuged at 5,000 gs for 10 min. The pellet was then dislodged by snapping the tube and 500 μl of QIAzol Lysis reagent (Qiagen) was added to the pellet. After the cells had been mixed for 1 min they were kept at −80°C until we started the preparation.

For RNA preparation samples were thawed at RT and the RNA purification performed as indicated by the manufacturer (see also Supplementary Materials and Methods).

### cDNA synthesis

cDNA preparation was conducted using a QIAGEN (RT^2^ First Strand Kit) kit, following the manufacturer's instructions; 2 μg of RNA were used for the reverse transcription (see also Supplementary Materials and Methods).

### Real-time PCR

In order to study the cell-cycle-regulated genes, a Real-Time PCR is performed using RT^2^ Profiler PCR Arrays (QIAGEN) in combination with RT^2^ SYBR Green Mastermix (PAMM-020ZC, QIAGEN) following the MIQE guidelines (Vandesompele et al., 2002; Taylor et al., 2010).

RT^2^ Profiler PCR Arrays in 96-well plates contains prime assays for 84 pathway focused genes and 5 housekeeping genes (which allows normalization of the data), a genomic DNA control (that detects non-transcribed genomic DNA contamination); 3 wells with reverse-transcription controls (important for testing the reverse-transcription reaction) and 3 wells of positive PCR controls with an artificial DNA sequence. The table of the genes used in the PCR array is reported in Supplementary Table S1.

In this experiment, 7 CTR and 4 HU samples were compared (see also Supplementary Materials and Methods).

CT values were normalized based on all plate analysis obtaining 5 most stable genes that were used as housekeeping genes: Cdk4 (Cyclin-dependent kinase 4), Cdkn3 (Cyclin-dependent kinase inhibitor 3), E2f3 (E2F transcription factor 3), Itgb1 (Integrin beta 1 (fibronectin receptor beta)), Shc1 (Src homology 2 domain-containing transforming protein C1) which result the 5 most stable within the plate. The algorithm identified these genes as the most stable housekeeping genes (Supplementary Table S2). Fold Change was obtained as 2^(−ΔΔCT)^. The data analysis web portal also produced a scatter plot, a volcano plot, a clustergram and a heat map.

### Cell cycle analysis by flow cytometry

Neurospheres from different samples (CTR and HU) at passages 3–10 were mechanically dissociated to single cells, and plated at 10,000 cells/cm^2^ in two 25 cm^2^ flasks in order to have two-time points at 3 and 5 days of culture and kept at 37°C and 5% of CO_2_.

After 3 or 5 days, cells were harvested, centrifuged and resuspended in GM buffer; fixation was obtained by adding ethanol to the final concentration of 70% and left overnight (ON) at 4°C. Subsequently, cells were washed with PBS 1X containing FBS at 5% and centrifuged at 664 g for 4 min at 4°C. The supernatant was discarded and the pellet was resuspended in PBS 1X, 60 μg/ml RNase A, 25 μg/ml propidium iodide at a final concentration of 25 μg/ml and 0.04% NP40 (for membrane permeabilization). After incubation ON at 4°C in the dark, samples were acquired with a FacsVerse flow cytometer and results were analyzed using the FACSuite software (BD Biosciences).

### Metabolism study

#### MTT assay

Cells were plated onto Cultrex (Tema Ricerca, Italy)-coated 96-well plates at a concentration of 10,000–15,000 cells/well in 200 μl of the PM at 37°C 5% CO_2_.

One day after plating and 1 h before collection, the tetrazolium dye 3-(4,5-dimethylthiazol-2-yl)-2,5-diphenyl-2H-tetrazolium bromide (MTT) (5 mg/ml in PBS; Sigma) was added to the medium (final concentration of 500 μg/ml).

Following 1 h of incubation at 37°C, the PM was discarded and cells were lysed by adding 50 μl of DMSO. After 15 min at RT, MTT reduction was measured spectrophotometrically by an ELISA reader (Sunrise Tecan) at a wavelength of 550 nm. As background value, we carried out an MTT analysis 2 h after plating (this time was the minimum necessary for the attachment of the cells on the well).

#### Lactate production

The analysis was performed using a L-Lactate Assay Kit II (Eton Bioscience, San Diego, CA, US) following the manufacturer's instructions (see also Supplementary Materials and Methods). The standard curve was made using a L-lactate standard provided in the kit and the measure of the absorbance was performed at 570 nm in a microplate reader (Sunrise Tecan). The calculations were made correcting for the background by subtracting the value of the blank from all sample readings.

#### Statistical analysis

All data were normally distributed according to the Shapiro-Wilk normality test. All the data are expressed as mean (AV) ± Standard Error Mean (SEM). Data were evaluated by the unpaired *t*-test. Results were considered statistically significant at p< 0.05 (see also Supplementary Materials and Methods).

## Results

### The sub-ventricular zone of the motor deprived animal shows fewer proliferating cells than in control animals

**Figure 1:**
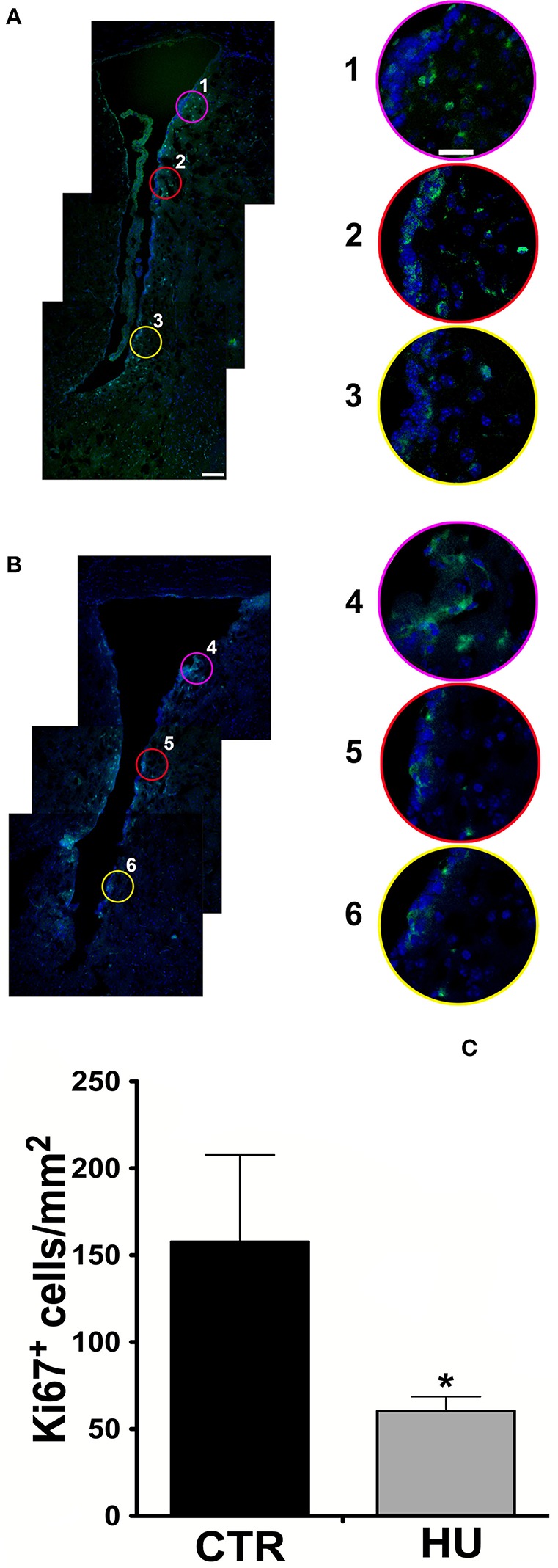
Confocal analysis of 10 μm coronal sections at the bregma level. Staining for Ki67 (Alexa 488 Goat anti-rabbit antibody), nuclei are stained with DAPI in blue **(A)** CTR mouse SVZ (1, 2, 3 are enlargements of the upper, medium and lower region of the SVZ) **(B)** HU mouse SVZ (4, 5, 6 are enlargements of the upper, medium and lower region of the SVZ). The border in pink, red and yellow of the enlargements corresponds with the circle indicated in the lower magnification figure **(A,B)**. **(C)** The number of Ki67 positive cells per mm^2^ in the area described in material and methods. Values represent means ± SEM. We analyzed the statistical differences with the one-tailed unpaired *t*-test *p* < 0.0365. Significance symbols: ^*^p < 0.0365. Scale bar 100 μm in **A** is also representative for **B**; scale bar 25 μm in 1 is representative of enlargement 2, 3, 4, 5, and 6).

In order to assess the impact of the hindlimb suspension on the number of SVZ proliferating cells, we analyzed the SVZ of the HU and CTR animals comparing the proliferation capability measured as an expression of Ki67 marker as shown in Figures 1A,B.

Following the work of Yasuhara et al. (2007), in order to take a snapshot of the changes in proliferating cells under movement constraint, Ki67 positive cells were measured in the SVZ and in the parenchyma of the ventral part of the SVZ within 300 μm of the ventricle, where the most proliferating cells are present, over an area of 0.5 mm^2^. For this study, more than 3 animals were used for the groups (3 CTR and 4 HU in the first experiment and 6 CTR and 4 HU in a second experiment) and we found that the suspended animals had 70% fewer proliferating cells than CTR mice (Figure 1C). Statistical analysis was performed by means of the one-tailed unpaired *t*-test.

The images were zoomed to discriminate the signals and all the positive Ki67 cells residing in the area were counted. The average results obtained were 157.7 ± 50.0 cell/mm^2^ for CTR and 60.4 ± 8.30 cell/mm^2^ for the HU *p* < 0.0365. The result (of the CTR group) is in agreement with those obtained from other authors in mice (Azim et al., 2012), as we extrapolated the number of cells from the heat map, and in rats (Yasuhara et al., 2007).

### Neural stem cells obtained from HU mice show a lower proliferation capability than CTR mice

**Figure 2:**
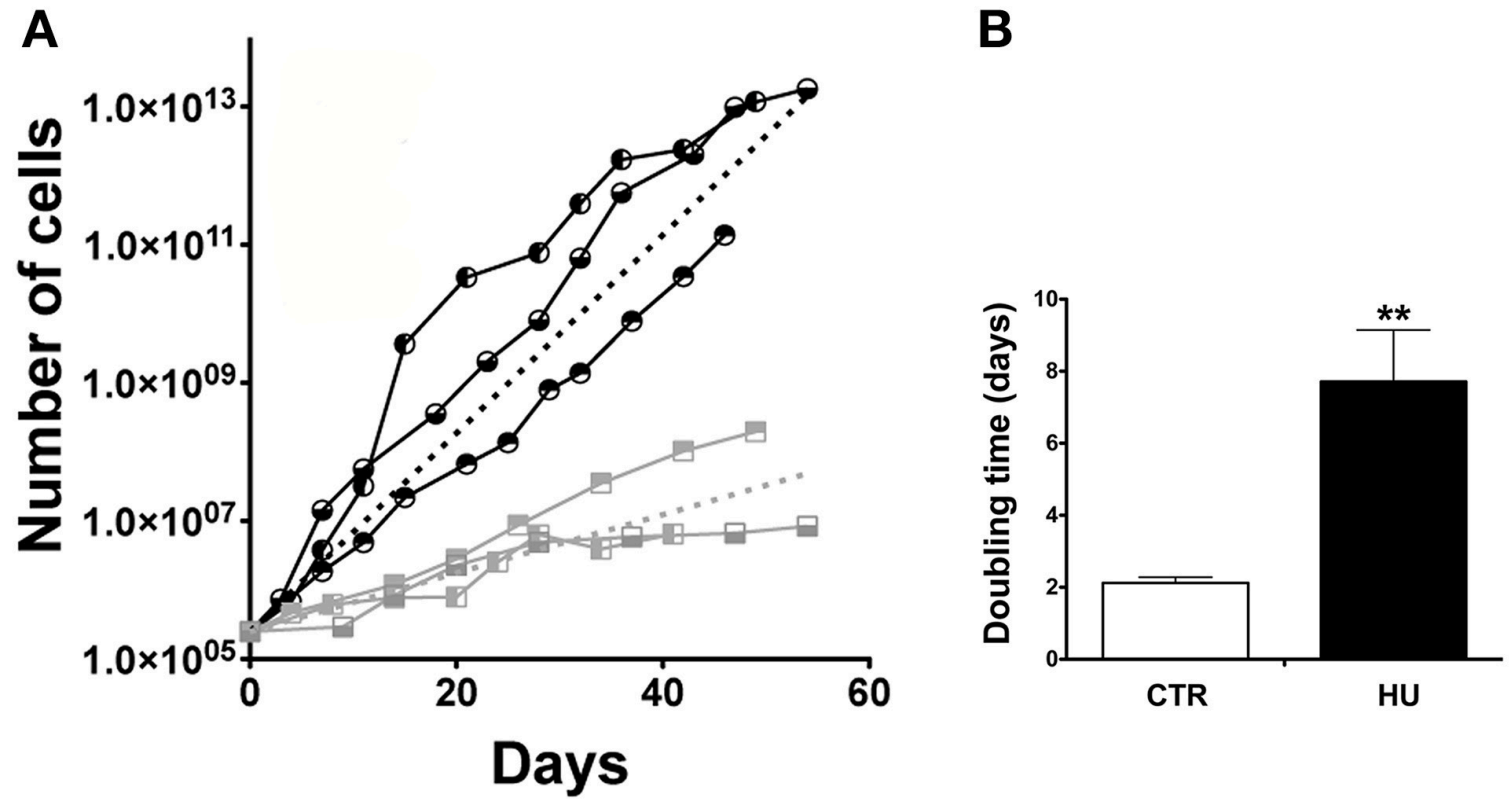
Growth curve and doubling time. **(A)** Proliferation capabilities comparison between CTR and HU samples. Each node of the curve represents a passage. The analysis was performed for 60 days of growth. 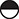 CTR1; 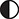 CTR2, 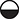 CTR3; 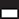 HU1; 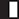 HU2; 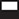 HU3. Dashed lines represent the average of CTR (black) and HU (gray). The average curves were built as the average of the slope of the CTR curves and HU curves with the constraint that the curve has value 250,000 at day 0. **(B)** Doubling time of proliferation expressed in days was calculated as the average of the doubling time of the single samples by means of non-linear fit regression. Black CTR, Gray HU. ^**^p < 0.0089.

We were able to isolate NSCs from both groups of 4-month old mice: those that were suspended by the tail and the control mice, which were free to move in their cage. Cultures from HU animals emerged more slowly than from the CTR (the morphology of the neurospheres was comparable) and their ability to proliferate in the following passages (by means of mechanical dissociation) displayed a less steep slope (Figure 2A). The average slopes, calculated from the curves of the HU and the CTR, were significantly different. The curves were calculated for each sample; and a non-linear regression (using the Prism program) was made, obtaining a slope (the starting point was restrained to 250,000 cells). The analysis showed that the average slope and the Standard Error Mean (SEM) were 0.33063 ± 0.0237 *N* = 3 (Average ± SEM) for the CTR and 0.09753 ± 0.0208 *N* = 3 for HU. Statistical analysis was performed using the one-tailed unpaired *t*-test. The values were significantly different *p* < 0.0009 (Figure 2A). In addition, the doubling time of the CTR samples (passage (P) 3–12) was 2.120 ± 0.16 days *N* = 3 whereas the HU samples (P3–12) had a doubling time of 7.173 ± 1.434 days *N* = 3, *p* < 0.0089 (Figure 2B); the analysis was performed using the one-tailed unpaired *t*-test.

**Figure 3:**
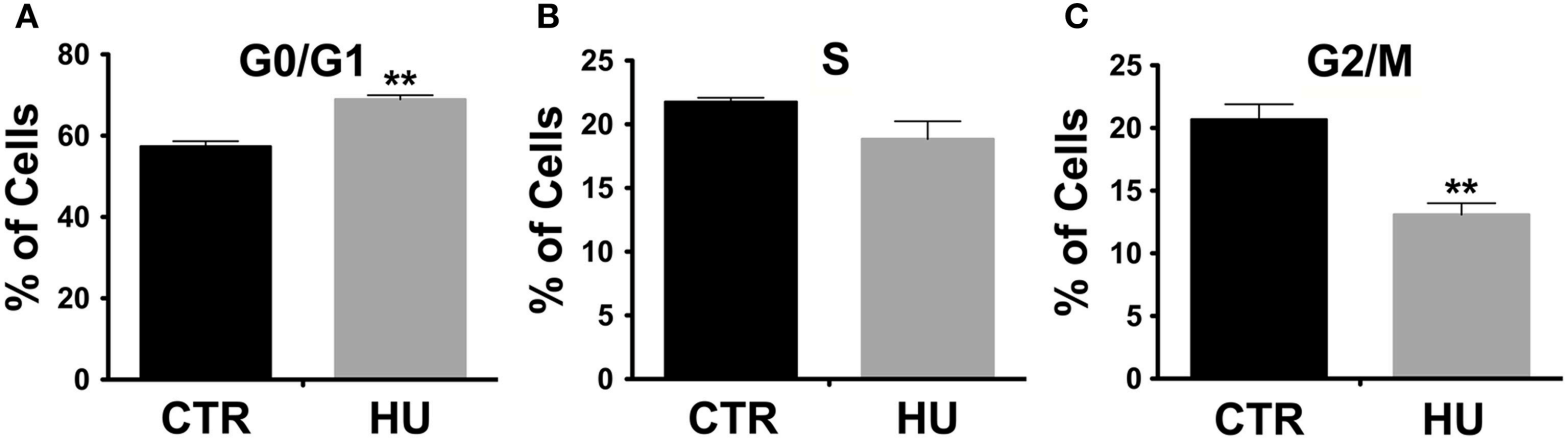
Cell cycle analysis by flow cytometer. A significant increase in cell numbers is present in G0/G1 in HU samples ^**^p < 0.0022 **(A)**; meanwhile a mild decrease in phase S **(B)** and a significantly lower percentage in G2/M **(C)** are observed. ^**^p < 0.0075 **(C)**. Black CTR, Gray HU.

The observed difference in proliferation (in a similar range of passages) was in accordance with the alteration in the cell cycle. Indeed, cell cycle analysis, performed by the flow cytometer, showed a significant increase in cell numbers in G0/G1 in HU samples (Figure 3A): 68.95 ± 1.013 for HU and 57.36 ± 1.303 for CTR, *p* < 0.0022. Meanwhile, a mild (not significant *p* < 0.1121) decrease was observed in phase S (Figure 3B) and a significantly lower percentage in G2/M 20.68 ± 1.212 for CTR and 13.09 ± 0.9132 for HU, *p* < 0.0075 (Figure 3C) (see also Supplementary Figure S1). For these experiments we used 4 samples for CTR and HU, the experiment was repeated 3 times and the analysis was performed using the two-tailed unpaired *t*-test.

No significant differences were detected at the level of apoptosis and senescence of the HU and CTR NSCs (see Supplementary Data). On the other hand, the number of cells expressing Nestin, Ki67, and GFAP was significantly altered in HU derived NSCs (see Supplementary Figure S3 and Supplementary results).

### Neural stem cells obtained from HU mice show lower differentiation and maturation capabilities than CTR mice

**Figure 4:**
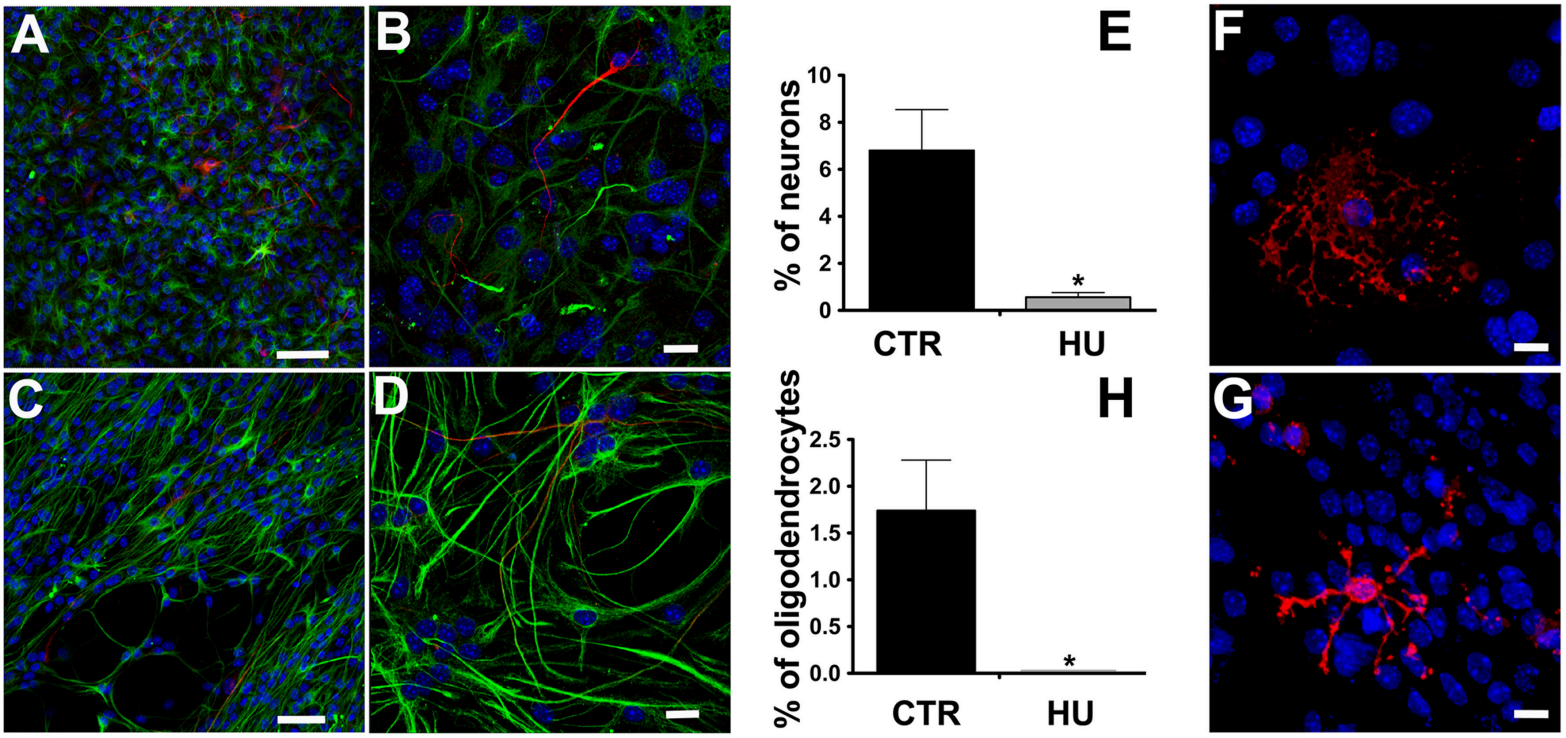
Immunostaining of the differentiated cells. β-tubulin III (red, Alexa 555 Goat anti-mouse antibody) and GFAP (green, Alexa 488 Goat anti-rabbit antibody), nuclei (blue, DAPI) staining. **(A**,**C)** Bar 50 μm. **(B,D)** higher magnification, Bar 20 μm. **(E)** Quantitative analysis of β-tubulin III marker expression in CTR (*n* = 4) and HU (*n* = 4), ^*^p < 0.05. O4 (red, Alexa 555 Goat anti-mouse antibody), nuclei (blue, DAPI) staining **(F)** CTR, **(G)** HU. **(H)** Quantitative analysis of O4 marker expression in CTR (*n* = 4) and HU (*n* = 4), ^*^p < 0.05. Bars 20 μm.

We observed that the differentiation of NSCs obtained from suspended mice was impaired, showing a significantly lower number of β-tubulin III positive cells than in CTR NSCs and a co-expression of glial fibrillary acidic protein (GFAP) (Figures 4A–E), suggesting an incomplete differentiation and/or maturation of the NSCs which, most likely, do not achieve mature neuronal electrical membrane properties. The percentage of differentiated neurons was 6.8 ± 1.74 (average ± SEM) *N* = 4 in the CTR and 0.56 ± 0.2 *N* = 4 *p* < 0.0119 in the HU samples. Similarly, the HU cultures showed a lower ability to produce oligodendrocytes (Figures 4F–H). The percentage of the differentiated oligodendrocytes also dropped in this case, from 1.74 ± 0.54 *N* = 3 in the CTR to 0.00 ± 0.01 *N* = 3 *p* < 0.0322 in the HU samples. The statistical analysis of changes in differentiated cells was performed using the two-tailed unpaired *t*-test.

### Suspended animals showed alterations in cell cycle gene expression

**Figure 5:**
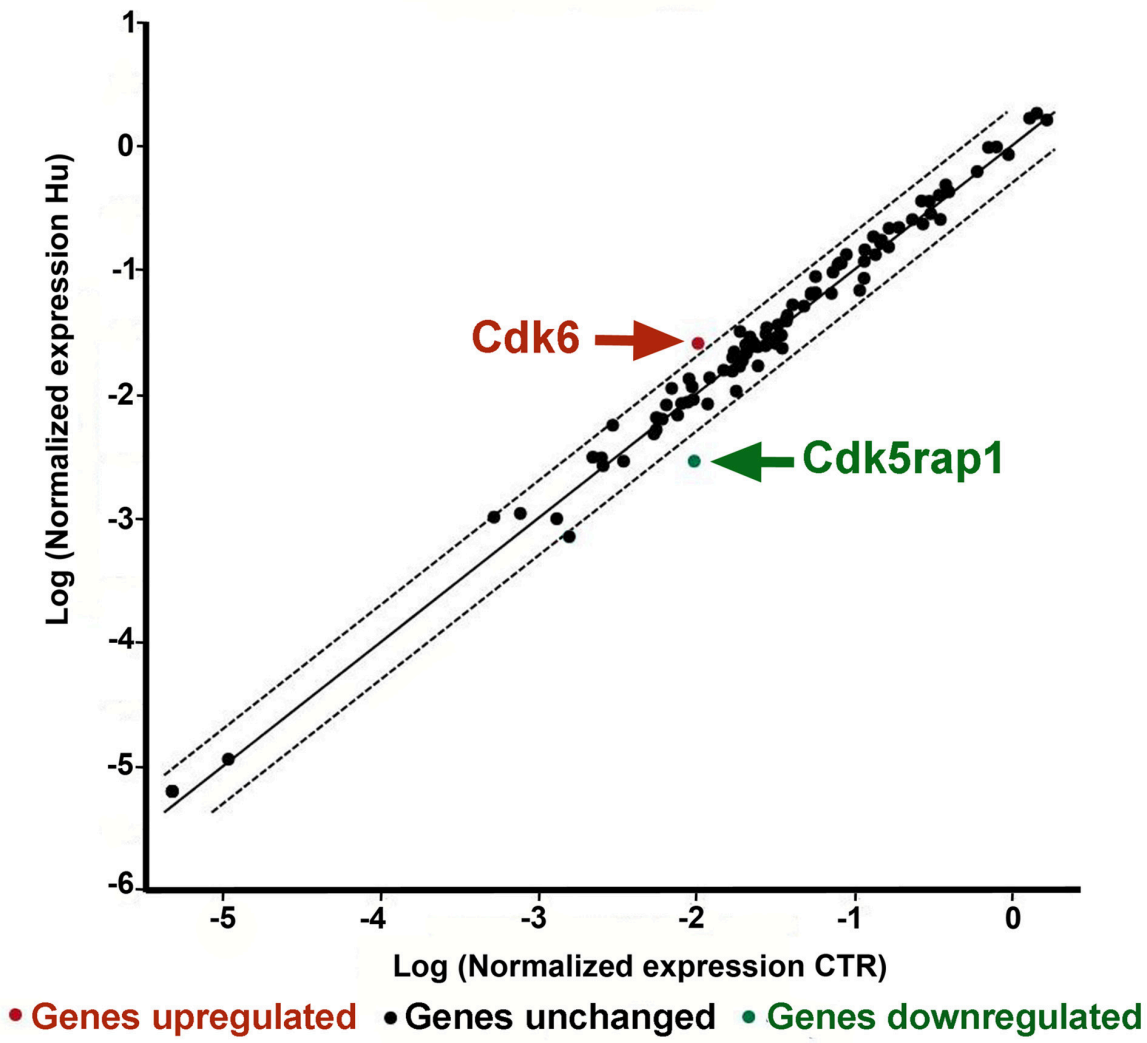
PCR Array analysis of CTR and HU samples. The scatter plot analyzes the normalized expression of all genes on the array between the two groups (CTR and HU) by plotting them against one another and indicating large gene expression alterations. The central line indicates unchanged gene expression whereas the dotted lines show the selected fold regulation threshold. Data points beyond the dotted lines in the upper left and lower right sections exceed the selected fold regulation threshold. The two genes that were observed to be significantly different between CTR and HU are indicated by arrows. (Controls = CTR, *n* = 7, and Hindlimb Unloading = HU, *n* = 4).

Two genes were found to be significantly different between CTR and HU samples: Cdk5 regulatory subunit-associated protein 1 (Cdk5rap1) with a fold regulation of −3.53 *p* < 0.005624 and cyclin-dependent kinase 6 (Cdk6) with a fold regulation of 2.38 and *p* < 0.021001 Figure 5 (Supplementary Figure S2 and Supplementary Table S3). Another gene, cyclin-dependent kinase inhibitor 2A (Cdkn2a), showed a fold change regulation of more than 2 but this difference was not significant (Supplementary Figure S2 and Supplementary Table S3).

Other genes showed an altered expression between HU and CTR but these differences were either lower than 2-fold or not significant.

Statistical analysis was accomplished by means of the two-tailed unpaired *t*-test.

### Metabolic alteration in movement-restrained mice

It is known that NSCs have a mainly glycolysis-based metabolism, which shifts to oxidative metabolism during their differentiation. In order to evaluate and compare the viability and the metabolism of the two groups of NSCs (CTR and HU), we performed an MTT assay and analysis of lactate levels produced by the cultures.

**Figure 6:**
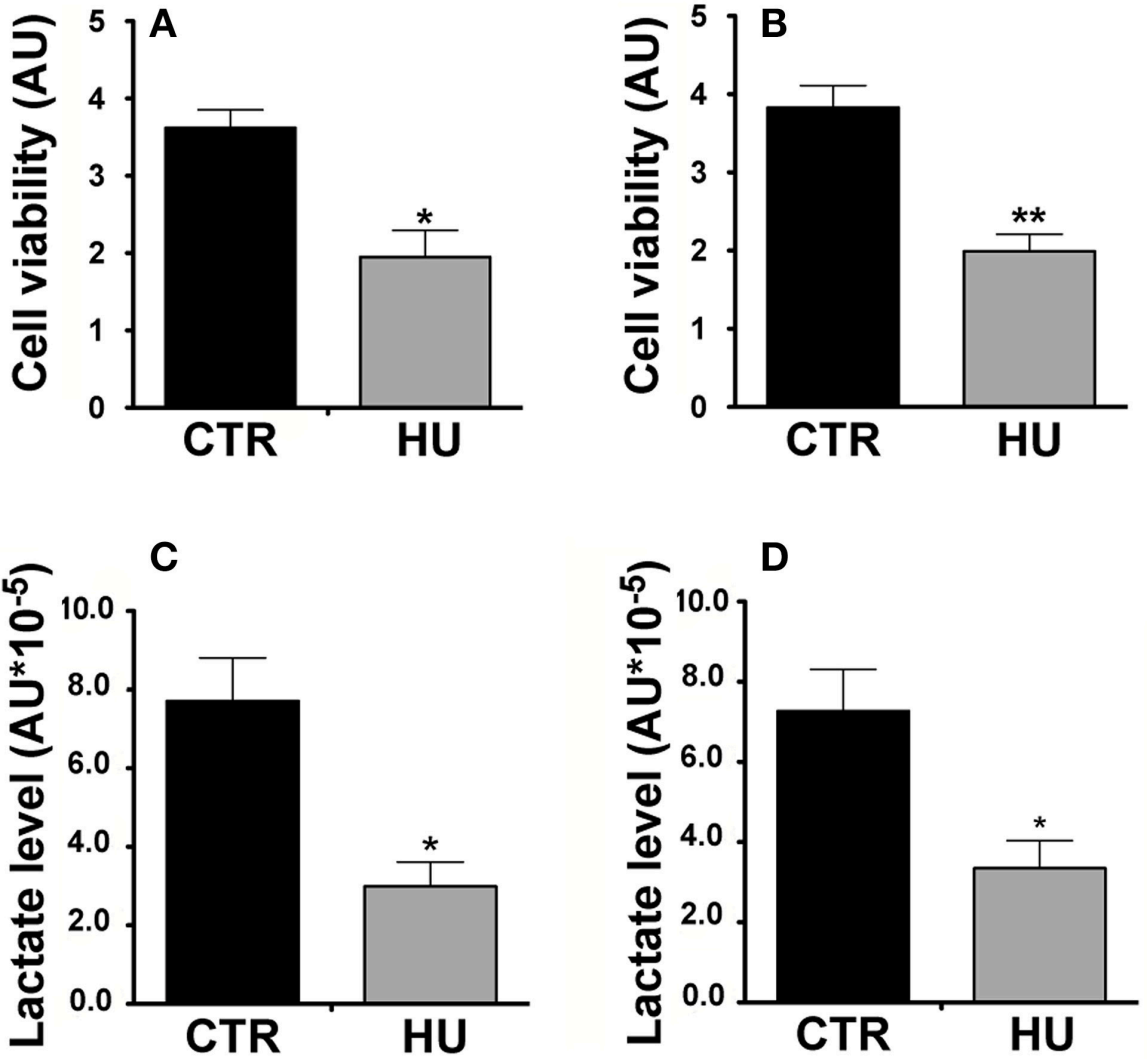
Metabolic activity of HU and CTR NSCs. MTT Assay. **(A)** Cell viability expressed in arbitrary units (AU) after one day of *in vitro* culture ^*^p < 0.0364. **(B)** Cell viability expressed in AU after three days of *in vitro* culture ^**^p < 0.0017. Black = CTR; gray = HU. **(C)** Lactate level: Relative lactate production expressed in AU ^*^10^−5^, 4 h of *in vitro* culture ^*^p < 0.0142. **(D)** Relative lactate production expressed in AU ^*^10^−5^, 18 h of *in vitro* culture ^*^p < 0.0275. Black = CTR; gray = HU.

For the MTT assay, in order to obtain the starting point of metabolic activity, we first measured the relative MTT level after cell adhesion on the wells (previously coated with Cultrex, Tema Ricerca, Italy). After 1 or 3 days, the experiment was performed and the measurements obtained were subtracted from the background (measured in a well with no cells) and scaled as a function of the baseline obtained at day 0. The cell viability of the HU cells (expressed as arbitrary units) was significantly lower than that of the CTR (the former 40% of the latter), (*p* < 0.0364) at one day of culture (Figure 6A) and 52% (*p* < 0.0017) at 3 days of culture (Figure 6B). The experiments were performed three times using at least 5 samples for the CTR and 4 samples for HU.

MTT assay statistical analysis was performed using the two-tailed unpaired *t*-test.

Our results confirmed the lower metabolic capability of the HU samples; indeed after 4 h of culturing HU samples showed 61.2% less lactate than CTR samples (*p* < 0.0142) (Figure 6C) whereas after 18 h of culturing the HU samples level was 46.1% of that of the CTR, *p* < 0.0275 (Figure 6D). Statistical analysis was accomplished by means of the two-tailed unpaired *t*-test.

## Discussion

A severe reduction of movement is present in many different pathologies such as spinal cord injury, spinal muscle atrophy, amyotrophic lateral sclerosis, and multiple sclerosis, and can be due to impairment of the skeleton muscular apparatus or of the central and peripheral nervous system. This condition is also common in bedrest patients (due to a wide range of pathologies) or in astronauts who experience low gravity for prolonged periods. Reduction of movement or gravity stimuli exerts an important effect on the human body, altering the activity of many organs including the brain. To study these effects, we took advantage of the hindlimb unloading protocol (Morey et al., 1979; Desaphy et al., 2005), which reproduces the reduction of movements or the absence of gravity in space.

Our previous experience with HU mice, which were used for the present and other studies (Brocca et al., 2010; Desaphy et al., 2010; Maffei et al., 2014; Cannavino et al., 2015), indicates that HU can produced significant stress in about 6% of cases and during the first 3 days of suspension (Kawano et al., 1994; Morey-Holton and Globus, 2002; Basco et al., 2010; Gaignier et al., 2014; Mysoet et al., 2014), after which the levels of stress, serum, and urine corticosterone measured, returned to the normal ones of the non-suspended animals. Indeed, it had already been demonstrated (Kawano et al., 1994) that in 5-week-old rats corticosterone urinary excretion in the control group was 87.4 ± 12.8 ng/day and did not change throughout the unloading time. A significant increase (more than 3-fold) in corticosterone excretion was found on days 1 and 3 during the suspension before returning to control levels, this time is little longer in older rats (Kawano et al., 1994). Meanwhile, the number of proliferating cells in rat neurogenic areas was not affected by adrenalectomy, the treatment of these animals with corticosteroids induced a significant increase in the hippocampus but not in the SVZ (Alonso, 2000). Other authors (Lee et al., 2016) demonstrated that chronic injection of corticosterone, for 28 days, can significantly reduce the cell proliferation and the differentiation in the SVZ. However, this treatment mimics a very long stress condition that is not analogous to our model. Indeed, even after 21 days, HU mice did not show any significant difference in corticosterone plasma level respect to control mice (Gaignier et al., 2014).

The thymus and the spleen weight (normalized by body weight) and the number of nucleated cells of the spleen were demonstrated not statistically different between CRT and HU (Gaignier et al., 2014). In our experience, we found no difference in the weight of the adrenal gland (normalized by body weight) between suspended and CTR mice (Bottai, Recchia, and Canepari unpublished results).

All these data indicate that the suspended animals, which can freely explore the cage without climbing, underwent little stress and most likely the changes in NSCs characteristics are due to the reduced overall amount of movements, by selective disuse of the hindlimb muscles. Although HU mice back limbs are free to make voluntary movements through the full range of motion, limb muscles are not exposed to the weight load and are lacking an active muscle force and strength development.

Many hypotheses can be drawn to explain the role of HU on NSCs properties. The reduction of movement of hindlimbs could induce an alteration in afferent signaling and feedback information from intramuscular receptors, to the cerebral cortex, due to a modification of the reflex organization in hindlimb muscle groups (D'Amelio et al., 1998). Similarly, a prolonged unilateral lower limb suspension, in humans, induces a variation of the nervous system plastic properties (Clark et al., 2006). On the site of neurotransmitters, it was demonstrated that 28 days of HU exposure in rats induced changes in the memory function and spatial learning that were related to protein expression changes (75 proteins were over-expressed and 72 down-regulated; Wang et al., 2017). In particular, the expression of Glutamate receptor (GluR) 1, GluR4, and the level of glutamate were up-regulated (Wang et al., 2017). On the other hand, the concentration of serotonin, dopamine, GABA, and epinephrine was reduced respect to control rats (Wang et al., 2017). Others studies indicated that HU-induced mRNA levels alterations of trophic factors such as NGF and BDNF in the somatosensory cortex (Dupont et al., 2005). In addition, many factors, such as cytokine and peptides, but also metabolites, exosomes, and miRNA, are released in the circulating system by the muscle during its activity allowing the possibility to influence other tissue (i.e., bone, liver, adipose tissue, cardiovascular system, brain) with autocrine, paracrine, and endocrine effects (Pedersen, 2011; Giudice and Taylor, 2017; Pourteymour et al., 2017; Safdar and Tarnopolsky, 2018; Whitham et al., 2018). Finally, other authors have previously demonstrated that the inhibition of bone formation, induced by unloading, is a consequence of the hindlimb elevation independently of the glucocorticoids since plasma corticosterone levels are not significantly different between normally loaded and unloaded rats and adrenalectomy had no protective effect on bone loss in unloaded animals (Halloran et al., 1988).

In our previous experience, where mice refused to eat during the first 2 days of suspension they were unfastened on the second day and discarded from the study. In the current study, none of the animals were discarded. Our previous observations indicated that the body weight of the animals that undergo suspension have a slight but significant decrease in their body mass (Desaphy et al., 2010), whereas other researchers even with prolonged suspension did not find differences compared to control group (Colaianni et al., 2017).

During the unloading experiment, mice were monitored daily for appropriate food and water intake, urination and defecation and normal grooming behavior, which were observed to be statistically similar between the two groups, indicating that they experienced very little stress. The weights of the antigravity muscles (gastrocnemius and soleus) were decreased significantly (respectively by 10 and 20%) after unloading, as already demonstrated in previous work of our group (Brocca et al., 2010) and from other groups (Hanson et al., 2013).

Our study adds more information for a better understanding of the role of movement reduction in NSCs features. It is known that physical inactivity is a risk factor for Alzheimer's disease (AD) (Hashimoto et al., 2017) since hippocampal atrophy was associated with the AD. In a recent meta-analysis, Guure and collaborators found that physical activity is more protective against AD than all the other forms of dementia (Guure et al., 2017).

Physical activity induces an increase in the hippocampal volume and ameliorates the neurogenesis (Bednarczyk et al., 2009) most likely via the augmentation of the blood flow (Cass, 2017). Consistently with this, the level of VEGF in the plasma of suspended rats decreases after 14 days of treatment, whereas it remains unaltered in the brain (Yasuhara et al., 2007) and in the soleus muscle (Wagatsuma, 2008). Long suspension (4 weeks) induced an inflammatory response in the common carotid artery of rats exposed to simulated microgravity, indicating that the inflammatory response may be a cellular mechanism that is responsible for the arterial remodeling during exposure to simulated microgravity (Liu et al., 2014). Prospective studies indicate that physical inactivity is one of the most frequent avoidable risk factors for developing AD. Moreover, elevated physical activity levels are associated with a lower risk of AD. The AD patient who undertook exercise training showed decreased neuropsychiatric symptoms, improvement in cognitive function, and a slower decline in the activities of daily life (Cass, 2017). After exercise AD patients regained some of their brain capabilities following appropriate and controlled physical training (Okonkwo et al., 2014).

We decided to study SVZ because its role in neurogenesis in the adult human might—albeit not comparable, in term of activity, with rodents—have a major impact on human brain health. It is already known that neuroblasts derived from SVZ migrate through the rostral migratory stream system into the olfactory bulb (Sanai et al., 2004; van den Berge et al., 2010). However, there is another major migratory pathway of immature neurons destined for the prefrontal cortex in infants (Sanai et al., 2011) and it was found, by measuring the levels of nuclear bomb test-derived ^14^C in genomic DNA (Bergmann et al., 2012), that there is insignificant neurogenesis in the human olfactory bulb. Surprisingly it was determined that there is a postnatal cell turnover in the striatum of adult humans (Ernst et al., 2014) that the authors suggested derives from the SVZ. Despite this evidence, there are limited hints about the role of exercise on neurogenesis in SVZ which impact remains debated (Brown et al., 2003; Bednarczyk et al., 2009; Blackmore et al., 2012; Chae et al., 2014). On the contrary, the positive role of exercise, especially in the case of previous brain impairment, is well assured (Lee et al., 2016; Mastrorilli et al., 2017). Finally, the impact on the hippocampus is more extensive and established (Lee et al., 2016; Mastrorilli et al., 2017; Snyder et al., 2017; Bouchard-Cannon et al., 2018; Firth et al., 2018; Masrour et al., 2018).

This range of evidence prompted us to carry out an *ex vivo* analysis demonstrating that, as already showed in the rat hippocampus model (Yasuhara et al., 2007), hindlimb unloading exerts a reduction in proliferating Ki67 (an endogenous marker of cell proliferation, expressed in the nucleus in all phases of the cell cycle except the resting phase, that is associated with, and necessary for, cellular proliferation) positive cells in the SVZ (Figure 1).

This first step allowed us to predict some alteration in NSCs attributes of the SVZ of the suspended animals; we then proceeded with the preparation of NSC cultures from this region.

We were able to produce NSC cultures from the SVZ of the HU and CTR mice; we found that HU NSCs neurospheres were more difficult to obtain since they emerged more slowly than those from the controls. The morphology of the neurospheres of the two culture groups was similar, with no difference in shape or adhesion to the plastic of the flask. However, we noticed before doing any counting that the size of the neurospheres was clearly smaller in the HU group. The proliferation analysis was performed measuring the cell number in the exponentially growing phase for many passages. This procedure showed that the proliferation capability of the HU derived SVZ NSCs was impaired, confirming the result obtained in *ex vivo* analysis (Figure 2). We analyzed the expression level of epitopes routinely detected in NSCs such as Nestin, GFAP, SOX2 (Kuhn et al., 2016), GLAST (Gubert et al., 2009), and Ki67, that is expressed in proliferating cells in all active phases of the cell cycle. The number of cells expressing Nestin, Ki67, and GFAP was significantly reduced in HU derived NSCs. The diminished number of Ki67 positive cells supports our results on NSCs proliferation (Figure 2). A decrease in Nestin and GFAP positive cells (Supplementary Figure S3) could be related to fewer committed glial cells that led to less glial differentiation as also shown in Figure 4. On the contrary, Sox2, that is considered a more trustworthy stem cell marker (Sarkar and Hochedlinger, 2013), did not show any changes in its expression. This result can be explained considering that we evaluated the number of positive cells, which is correlated with the change in cell cycle kinetics that we observed (Figure 3), rather than a decrease in the level of expression of these markers per cell. Further analysis will be necessary in order to understand these aspects.

The suspension induced an arrest of NSCs in the G0/G1 phase and a significant reduction in the G2/M phase (Figure 3), the G0/G1 arrest was also found in of rat bMSCs when exposed to simulated or real microgravity (Bradamante et al., 2014).

The effect of hindlimb unloading on the stereological parameters of the hippocampus and the motor cortex in male rats was recently assessed (see Supplementary discussion a).

Another interesting aspect is the potential reversibility of the alterations induced by adult NSCs in the SVZ, following a more or less long period of post-HU recovery. The recovery (after suspension) with or without exercise in rats were ineffective for the retrieval of proliferation and differentiation in SVZ (Yasuhara et al., 2007).

With regard to the metabolic capacity of the HU and CTR NSCs, it is recognized that intracellular pO_2_ values of 0.5 Torr or less occur in O_2_-limited oxidative phosphorylation and consequent lactate production and accumulation (Rogatzki et al., 2015). At low levels of oxygen, energy is provided by anaerobic metabolism, leading to the production of lactic acid (De Filippis and Delia, 2011). Stem cells, such as ESCs, HSCs, MSCs, and others, have immature mitochondrial morphology, a reduced oxidative capacity and enhanced anabolic levels in the glycolytic pathways (Folmes et al., 2012). Brain lactate is a major substrate for oxidative metabolism during development and it is selectively utilized as an anabolic reservoir for cell proliferation and differentiation. Furthermore, lactate is used actively by brain cells in culture (Medina and Tabernero, 2005). In this scenario, the results obtained from our work emphasize the involvement of metabolism alteration and NSCs properties during the reduction of motor activity.

The MTT assay has been widely used to assess cell viability; however, MTT effectively measures the metabolic activity of live cells, since it evaluates the capacity of cells to reduce the tetrazolium dye by functioning mitochondria. The levels of oxygen have been shown to affect NSCs characteristics during normal development, disease and culturing (Studer et al., 2000; Mohyeldin et al., 2010; Santilli et al., 2010). Since the NSCs have mostly a glycolytic metabolism, due to the low oxygen tension level (Zhang et al., 2015) the lower lactate level measured in HU NSCs indicates that these cells have a higher oxidative metabolism (Figure 6C,D), this was also demonstrated by other authors (Medina and Tabernero, 2005).

Contrary to what was expected, a gene, Cdk5 regulatory subunit-associated protein 1 (CDKrap1), was significantly reduced in HU samples relative to CTR (Figure 5). Cdk5rap1 was first found to be a negative Cdk5 regulator but in a subsequent study, Cdk5rap1 was found to be responsible for the post-synthetic conversion of the RNA modification N6-isopentenyladenosine (i^6^ A) into 2-methylthio-N6-isopentenyladenosine (ms^2^ i^6^ A). (Cdk5rap1) is responsible for 2-methylthio modifications of mammalian mitochondria (mt)-tRNAs for Ser(UCN), Phe, Tyr, and Trp codons (Wang et al., 2015; Wei et al., 2015). In breast cancer cells MCF-7 Cdk5rap1 paucity induces cell cycle arrest (Wang et al., 2015). The Cdk5rap1-KO mice are sensitive to stress-induced mitochondrial remodeling (see Supplementary discussion b).

Another gene which expression was altered in HU NSCs, compared to CTR NSCs is Cdk6.

On the basis of our findings, we can speculate that the low expression level of Cdk6 in NSCs from CTR animals correlates with their higher proliferation capacity, so the elevated level of expression of this gene in HU derived NSC should be implicated in their altered capabilities (Figure 5). Cdk6, acting as cell cycle kinase, promotes the progression to S phase, chromatin status, cell death, cell survival, and DNA repair interacting with cyclin D; moreover, behaving as transcriptional regulators, Cdk6 interacts with other proteins such as RUNX1, NF-kB, STAT3, and AP-1 regulating respectively differentiation, inflammation, cell cycle arrest, stress hematopoiesis and angiogenesis (Tigan et al., 2016). Cdk6 and cyclin D are very important drivers of tumorigenesis. Cdk6 can have a role as tumor suppressor reducing proliferation in lymphoid malignancies (Kollmann et al., 2013), or restrain the proliferation of breast cancer cells (Lucas et al., 2004). Further analysis will be necessary to depict this aspect.

These data, to our knowledge, are the first evidence of a correlation between changes in NSCs attributes after movement restraint, metabolism modification, and gene expression changes. Interesting, the ability of HU cells to maintain their altered properties for more than 10 passages of culture suggests that an epigenetic modification might be involved. In these regards, the fact that suspended animals underwent a stress during the first three days of the unloading period could explain the gene expression alteration in terms of epigenetic changes. However, the gene expression analysis we performed on the cell cycle genes showed no changes in genes that are usually affected during stress induction namely Cyclin D1 and Cyclin-dependent kinase inhibitor 1A (P21) (Juszczak and Stankiewicz, 2018) but rather other genes were altered.

Epigenetic modifications affect not only DNA and proteins, but also coding and non-coding RNAs. RNAs can be modified in more than 100 ways including *N*6-methyladenosine (m6A), *N*7-methylguanosine (m7G), m5C, pseudouridine, and queuosine (Jia et al., 2013). Many such modifications have a basic function in controlling aspects of RNA metabolism, such as splicing, transport, translation, and degradation. These active RNA modifications represent another level of gene regulation, termed “epitranscriptomics.” The discovery that Cdk5rap1 (which acts as methylthiotransferase in mitochondria RNA) was altered in its expression after motor deprivation is very intriguing in this context, and opens a new link between neurogenesis metabolism and cell regulation.

## Author contributions

RA and DB: designed research; RA, JP, MCo, NP, DR, MCa, and DB: performed research; RA, MCo, NP, RC, and DB: analyzed and interpreted data; RA and DB: wrote the paper; RA, JP, MCo, NP, DR, RC, RB, MCa, and DB: revised and approved the paper.

### Conflict of interest statement

The authors declare that the research was conducted in the absence of any commercial or financial relationships that could be construed as a potential conflict of interest.
