# Supplementary figures and images for: Reduction of Movement in Neurological Diseases: Effects on Neural Stem Cells Characteristics
Source: Front Neurosci. 2018 May 23;12:336. doi: 10.3389/fnins.2018.00336 (PMC5974544; doi:10.3389/fnins.2018.00336)

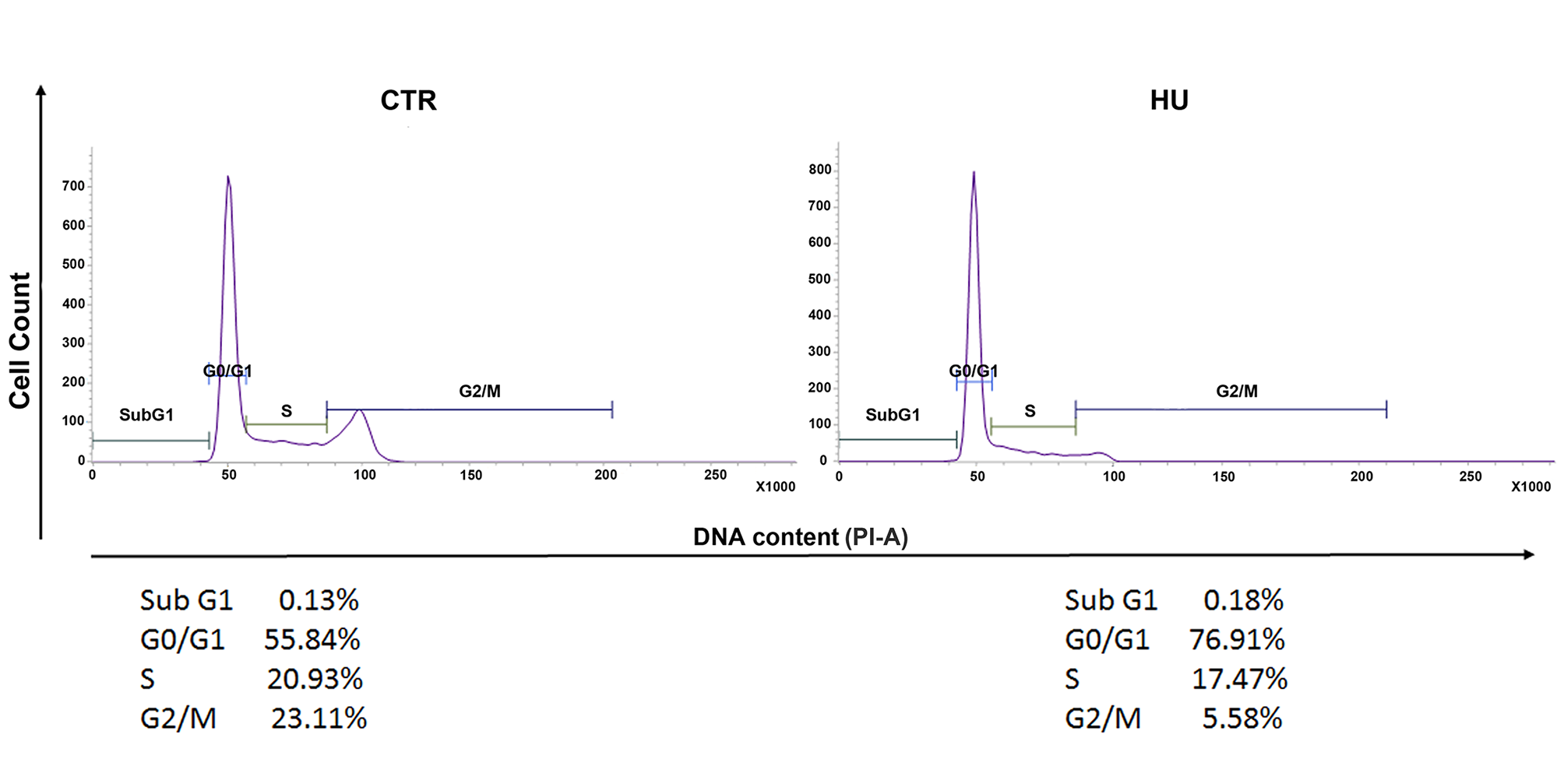

Supplement: Supplementary file 4 [file Image_1.TIF]

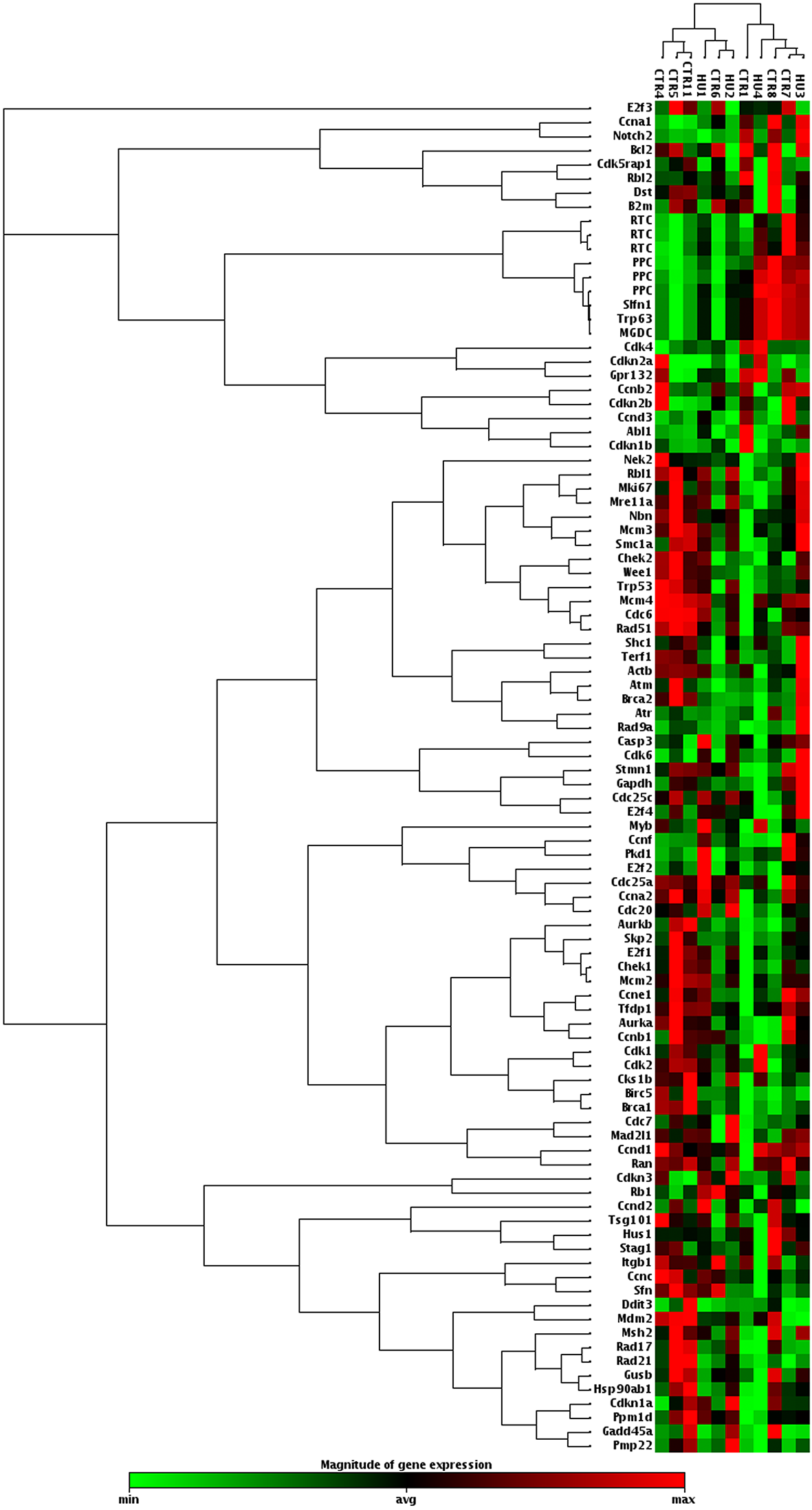

Supplement: Supplementary file 5 [file Image_2.TIF]

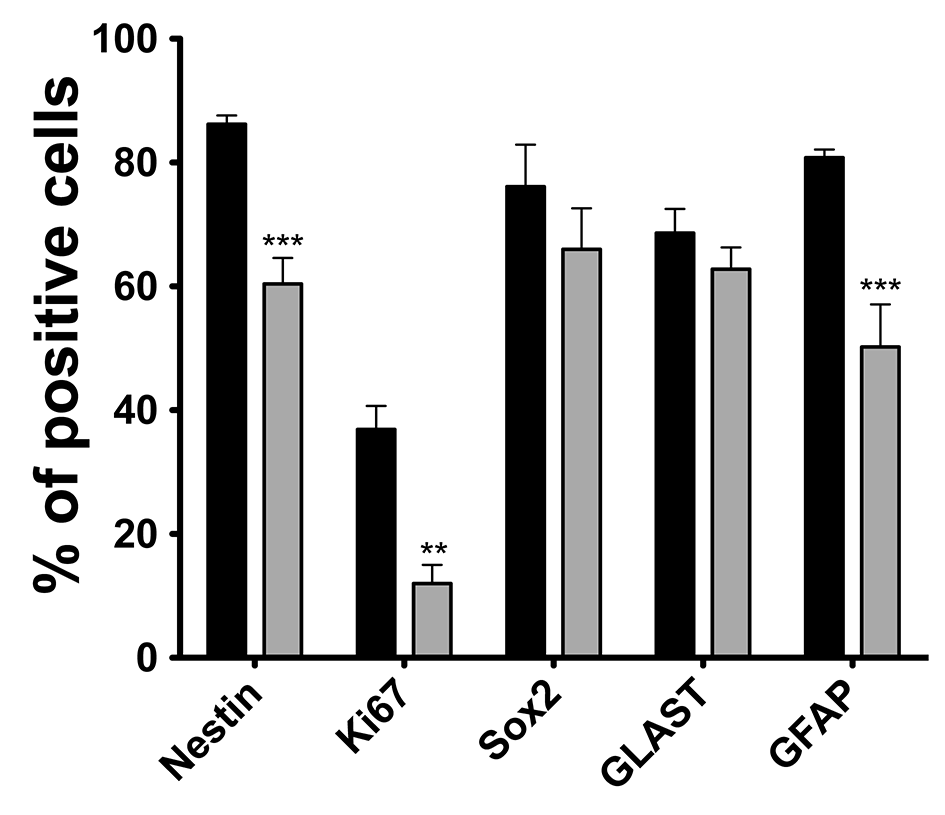

Supplement: Supplementary file 6 [file Image_3.TIF]
