# Supplementary material for: Reduction of Movement in Neurological Diseases: Effects on Neural Stem Cells Characteristics
Source: Front Neurosci. 2018 May 23;12:336. doi: 10.3389/fnins.2018.00336 (PMC5974544; doi:10.3389/fnins.2018.00336)
Supplement: Supplementary file 7 [file Data_Sheet_1.docx]

**Supplementary data**

Supplementary Materials and Methods.

*Solution composition*

PB 0,01M: NaCl 137 mM, KCl 2,7 mM, Na_2_HPO_4_ 10 mM, K_2_HPO_4_ 1.8 mM, pH 7.4;

PBS 1X: KH_2_PO_4_ 1.06 mM; NaCl 155.2 mM; Na_2_HPO_4_ 2.97 mM, pH 7.4;

GM buffer: PBS 1X, glucose 1mg/ml, ethylenediaminetetraacetic acid (EDTA) 0.2 mg/ml) and 2% Fetal Bovine Serum (FBS).

*Animal model*

The mice were examined daily over the entire HU period for behavior, cleanliness, the appearance of their fur and eyes and their food and water consumption. None of the animals had any rest periods during the suspension, none of the animals was removed from the study because they could not tolerate the suspension. Control mice (CTR) were housed individually in cages of the same size as the ones used for suspension (Tecniplast, Buguggiate, Italy).

*RNA isolation from proliferating Neurospheres*

As the first step, all samples were incubated at RT for 5 minutes, had 140 μl of chloroform added and immediately shaken for 15 seconds. After another incubation of 3 minutes at RT, samples were centrifuged for 15 minutes at 12,000 gs at 4 °C. The upper aqueous phase was transferred into a new collection tube where 1.5 volumes of 100% ethanol were added. The solution was mixed by pipetting up and down several times and transferred to an RNeasy Mini spin column (Qiagen), the subsequent steps as indicated by the manufacturer allowed us to produce a high quality (DNA free) RNA that was stored at -80 °C.

The RNA was quantified using a Thermo Scientific™ NanoDrop™ One Spectrophotometer; the ratios A260/A280 and A260/A230 were used as indicators for sample quality.

*Neurospheres staining*

Dissociated NSCs were plated in laminin-coated multiwell plates (48 wells per plate) where was previously put a round coverslip. In these conditions, cells grow in neurospheres structure but stay attached to the coverslip. When the neurosphere reach the appropriate size (0.1 mm) they are fixed in 1% formalin and stained for markers usually detected in NSCs such as Nestin (type VI intermediate filament protein, Immunological Science MAB-11004, 1:250), Ki67 (abcam ab92353, 1:200), SOX2 (Sex determining region Y-box 2, Immunological Science AB-81156 1:100), GLAST (GLutamate ASpartate Transporter, abcam ab416, 1:100 ), and GFAP (Immunological Science AB-10635, 1:250). The staining was performed as described in the materials and methods section with slight modifications, in particular, the time of incubation with the primary antibody was 48 hours. The secondary antibodies conjugated with fluorophores were Alexa-fluor 488 (Goat anti-mouse Immunological Sciences IS20010 for Nestin antibody) and Alexa-fluor 555 (Goat anti-rabbit Immunological Sciences IS20012 for Ki67, SOX2, GLAST, and GFAP) at a dilution of 1:800.

The positive cells were counted at a fluorescent microscope. Data were evaluated by two way ANOVA. Results were considered statistically significant at p< 0.05.

*cDNA Synthesis*

Starting from concentration values obtained from RNA quantification, 2 µg of RNA were used for the reverse transcription.

In order to eliminate all the genomic DNA traces, the kit includes a proprietary buffer (Buffer GE) for the treatment of the samples before performing the cDNA synthesis. The kit also contains a built-in external RNA control very important for the evaluation of the efficiency of the Real-Time PCR.

Briefly, the samples were treated for genomic DNA elimination and immediately placed on ice for at least 1 minute, then the reverse transcription was performed adding the transcription mix to each tube containing 10 µl of genomic DNA elimination mix and incubating at 42 °C for 15 minutes. The reaction was stopped by incubating the mix at 95°C for 5 minutes. Finally, 91 µl of RNase-free water were added to each tube, samples were mixed and cDNAs stored at -80° C.

*Real-time PCR*

CT values were exported to an Excel file to create a table of CT values; the CT cut-off was set to 35 and the fold regulation cutoff was set to 2. This table was uploaded on to the data analysis web portal at <http://www.qiagen.com/geneglobe>.The plate also contained internal control samples that allowed us to correct for the multiple comparisons.

A melting curve analysis was also performed to verify primers specificity. Fold change and regulation were calculated using the ΔΔCT method, in which ΔCT is calculated between gene of interest (GOI) and an average of reference genes (HKG), in a second step ΔΔCT was determined as ΔCT (Test Group)-ΔCT (Control Group). Fold Change was obtained as 2^(-ΔΔCT)^. The data analysis web portal also produced a scatter plot, a volcano plot, a clustergram and a heat map.

*Lactate production*

The analysis was performed using an L-Lactate Assay Kit II (Eton Bioscience, San Diego, CA, US) following the manufacturer’s instructions. Briefly, 15,000 cells were plated in a Cultrex (Tema Ricerca, Italy) coated well of a 48-well plate; when the cells reached 80% of confluence the proliferation medium (PM) was removed and substituted by a new PM. After 4 or 16 h the medium was harvested, centrifuged to remove cellular debris and used directly for the determination of the levels of lactate. The supernatant was diluted in a ratio of 1:2 for the determination. The samples were assayed in duplicate.

*Statistical analysis*

All data were normally distributed according to the Shapiro-Wilk normality test <http://scistatcalc.blogspot.it/2013/10/shapiro-wilk-test-calculator.html>*.* The group size was chosen on the basis of the results obtained in previous studies. The power analysis was conducted using “EEC Animal Experimentation Sample Size Calculator”, available from *The Laboratory Animal Services Centre at The Chinese University of Hong Kong* (Fitts, 2011). We calculate the number of animals necessary for each type of experiments. For instance, for the calculation of the animal number necessary for this neurogenesis study, we used the previous results obtained from Yasuhara and collaborators (Yasuhara et al., 2007), which were very highly correlated with our work, and a power analysis of 80%. An analogous analysis taking into account the results previously obtained from other authors (Fernandes et al., 2010) was used in order to study the changes in cellular metabolism.

With regard to the Real-Time PCR analysis, since the study was conducted on cell-cycle related genes by Real-Time PCR arrays (Qiagen) our calculation of the animal numbers necessary for the analysis was also based on the method described above, utilizing as previous work the paper from Ma and collaborators (Ma et al., 2015) in order that the power of our analysis would be 80 %.

*Apoptosis analysis by flow cytometry*

250,000 cells for each sample were plated in a T25 flask with the PM.

As soon the neurospheres reached the suitable dimensions, cells were harvested and transferred into 15 ml sterile falcon tubes. Cells were centrifuged at 100 gs for 10 minutes and the pellet was dissociated enzymatically using 200 μl of Accutase and incubated at 37°C for 15 minutes.

After the incubation, the enzyme was blocked with FBS, DMEM was added, and cells were centrifuged at 100 gs for 10 minutes. The supernatant was discarded and 200 μl of PBS 1X were added; afterwards, cells were counted and the total number of cells determined.

Afterwards, 800 μl of PBS 1X were added and cells centrifuged at 100 gs for 10 minutes. These steps were repeated twice but in the second step, cells were transferred to an Eppendorf tube.

The supernatant was then removed and samples were fixed with 4% formaldehyde for 20 minutes at RT. Permeabilization was performed by 10 mins incubation with a solution containing PBS, 0.5% BSA and 0.5% saponin at RT. Then cells were centrifuged and incubated for 1h at 4°C with the anti-cleaved caspase 3 antibody (Cell Signaling Technology, #9602). After the incubation, samples were analyzed by FACS (BD FACSVerseTM System).

*Senescence analysis*

The Senescence Cells Histochemical Staining Kit (SIGMA) is based on a histochemical stain for β-galactosidase activity at pH 6 which is present in senescence.

45,000 cells for each sample were plated in a 96-well plate previously coated with Cultrex ( Tema Ricerca, Italy) and left until 80% of confluence. The staining was performed as indicated by the manufacturer. Briefly, after removal of the growth medium, cells were washed twice with PBS 1X. The cells were fixed with the fixation buffer 1X (Fixation Buffer 10X contains 20% formaldehyde, 2% glutaraldehyde, 70.4 mM NaHPO_4_, 14.7 mM KH_2_PO_4_, 1.37 M NaCl and 26.8 mM KCl) and incubated for 6-7 minutes at RT. The cells were washed three times with PBS 1X, the staining mixture (composed of staining solution, reagent B (400 mM potassium ferricyanide), reagent C (400 mM potassium ferrocyanide), X-gal (5-bromo-4-chloro-3-indoyl-beta-D-galactopyranoside) solution and water) added to each well. The plate was then sealed with parafilm and incubated at 37°C without CO_2_ overnight. The blue-stained cells and the total number of cells were counted under the microscope and the percentage of cells expressing β-galactosidase was calculated.

Supplementary results

S1 Table

RT-PCR Profiler™ PCR Array Mouse Cell Cycle; list and position of the genes in the array.

S2 Table

Normalization analysis

This method allows selecting the internal control/housekeeping/normalization genes for the analysis. We used the arithmetic mean consistently across experiments. Only genes with small changes in their expression across different sample groups were selected (differences in CT values less than 1). Cdk4 (Cyclin-dependent kinase 4), Cdkn3 (Cyclin-dependent kinase inhibitor 3), E2f3 (E2F transcription factor 3), Itgb1 (Integrin beta 1 (fibronectin receptor beta)), Shc1 (Src homology 2 domain-containing transforming protein C1) were found to be the most stable genes to be used as housekeeping genes.

S3 Table

The table provides the fold regulation data used for the map and the comments associated with each one. A: This gene’s average threshold cycle is relatively high (> 30) in both the control and the test sample, and is quite low in the other sample (< 30).These data indicate that the gene’s expression is relatively low in one sample and most likely detected in the other sample, suggesting that the real fold-change value is at least as high as the calculated and reported the fold-change result. B: This gene’s average threshold cycle is relatively high (> 30), implying that its relative expression level is low, in both control and test samples, and the p-value for the fold-change is either not available or high (p > 0.05). C: This gene’s average threshold cycle is either not determined or greater than the defined cut-off (default 35), meaning that its expression was undetected, and the result not interpretable. Two genes (Cdk5rap1 and Cdk6) that showed a significant alteration of their expression, as described in the result section, are indicated in yellow.

Supplementary figures

S1 Fig.

A standard PI staining was used to measure cell cycle distribution based on cell DNA content. Graphs show the typical distribution of CTR (left panel) and HU (right panel) cells and represent three independent experiments with similar results. PI-A: Propidium iodide-Area.

S2 Fig.

The clustergram conducts non-supervised hierarchical clustering of the entire dataset to display a graphical representation of data where the individual values contained in a matrix are represented as colors. Dendrograms show co-regulated genes across groups or individual samples.

S3 Figure

Analysis of the immunostaining of the undifferentiated cells.

The graph represents the quantitative analysis of the number of cells that express markers detected in NSCs, in black are represented CTR and in grey HU. CTR (n=5) and HU (n=4), *: p<0.05.

The results indicated that Nestin, Ki67, and GFAP were expressed in a significantly lower number of cells in the HU derived NSCs respect to the control. ***=p<0.001; **p<0.01.

Supplementary discussion a

The effect of hindlimb unloading on the stereological parameters of the hippocampus and the motor cortex in male rats was recently assessed (Salehi et al., 2016). However, neither the volume of the motor cortex or hippocampus nor the numerical cell density of neurons in the six motor cortical layers I-VI were altered in HU animals with respect to the CTR, most likely because the analysis was macroscopic and only the overall volume was evaluated.

In contrast, we demonstrated that differentiation was impaired in NSCs obtained from HU animals, with a lower percentage of neurons and oligodendrocytes than those obtained from CTR mice (Fig. 4). Consistently with this finding, in the same mouse model altered differentiation was detected in hematopoietic and mesenchymal cells (Blaber et al., 2014) and in the SVZ the expression of doublecortin (a microtubule-associated protein expressed by neuronal precursor cells and immature neurons in embryonic and adult cortical structures) (Yasuhara et al., 2007). This aspect is present in many cell types, including erythrocytes, osteoclasts, osteoblasts, and megakaryocytes (Blaber et al., 2014). In addition, an *in vitro* model exposure to microgravity (spaceflight) demonstrated that embryonic stem cells (ES) showed a reduced differentiation capability since the expression of terminal differentiation markers for tissues derived from all three primary germ layers was inhibited (Blaber et al., 2015).

Supplementary discussion b

Some authors have speculated that these mitochondria alterations could be implicated in some neurological diseases, such as Parkinson’s disease, since Parkin, a ubiquitin protein ligase whose mutation seems to be responsible for Parkinson’s disease (Kitada et al., 1998), induces mitophagy translocating to mitochondria with low membrane potentials. Since ms^2^ modification deficiencies had a low basal mitochondrial membrane potential, it is possible that Parkin is recruited to mitochondria, inducing the accelerated mitophagy (Wei et al., 2015).

# Bibliography

Blaber, E.A., Dvorochkin, N., Torres, M.L., Yousuf, R., Burns, B.P., Globus, R.K., et al. (2014). Mechanical unloading of bone in microgravity reduces mesenchymal and hematopoietic stem cell-mediated tissue regeneration. *Stem Cell Res* 13(2)**,** 181-201. doi: 10.1016/j.scr.2014.05.005.

Blaber, E.A., Finkelstein, H., Dvorochkin, N., Sato, K.Y., Yousuf, R., Burns, B.P., et al. (2015). Microgravity Reduces the Differentiation and Regenerative Potential of Embryonic Stem Cells. *Stem Cells Dev* 24(22)**,** 2605-2621. doi: 10.1089/scd.2015.0218.

Fernandes, T.G., Diogo, M.M., Fernandes-Platzgummer, A., da Silva, C.L., and Cabral, J.M. (2010). Different stages of pluripotency determine distinct patterns of proliferation, metabolism, and lineage commitment of embryonic stem cells under hypoxia. *Stem Cell Res* 5(1)**,** 76-89. doi: 10.1016/j.scr.2010.04.003.

Fitts, D.A. (2011). Ethics and animal numbers: informal analyses, uncertain sample sizes, inefficient replications, and type I errors. *J Am Assoc Lab Anim Sci* 50(4)**,** 445-453.

Kitada, T., Asakawa, S., Hattori, N., Matsumine, H., Yamamura, Y., Minoshima, S., et al. (1998). Mutations in the parkin gene cause autosomal recessive juvenile parkinsonism. *Nature* 392(6676)**,** 605-608. doi: 10.1038/33416.

Ma, L., Liu, X., Wang, F., He, X., Chen, S., and Li, W. (2015). Different Donor Cell Culture Methods Can Influence the Developmental Ability of Cloned Sheep Embryos. *PLoS One* 10(8)**,** e0135344. doi: 10.1371/journal.pone.0135344.

Salehi, M.S., Mirzaii-Dizgah, I., Vasaghi-Gharamaleki, B., and Zamiri, M.J. (2016). Effect of hindlimb unloading on stereological parameters of the motor cortex and hippocampus in male rats. *Neuroreport* 27(16)**,** 1202-1205. doi: 10.1097/WNR.0000000000000675.

Wei, F.Y., Zhou, B., Suzuki, T., Miyata, K., Ujihara, Y., Horiguchi, H., et al. (2015). Cdk5rap1-mediated 2-methylthio modification of mitochondrial tRNAs governs protein translation and contributes to myopathy in mice and humans. *Cell Metab* 21(3)**,** 428-442. doi: 10.1016/j.cmet.2015.01.019.

Yasuhara, T., Hara, K., Maki, M., Matsukawa, N., Fujino, H., Date, I., et al. (2007). Lack of exercise, via hindlimb suspension, impedes endogenous neurogenesis. *Neuroscience* 149(1)**,** 182-191. doi: 10.1016/j.neuroscience.2007.07.045.
